# Effectiveness of Protection Motivation Theory on clinical factors, behavior change, and cardiovascular disease: An integrative review

**DOI:** 10.1016/j.ijnsa.2024.100267

**Published:** 2024-11-13

**Authors:** Maryam Ghasemiardekani, Virginia Plummer, Louisa Lam, Biswajit Banik, Wendy Cross

**Affiliations:** a School of Health and Wellbeing, Federation University Australia, Victoria, Australia; b Federation University Australia, Victoria, Australia; c School of Health and Wellbieng, Federation University Australia, Victoria, Australia; d School of Nursing, Midwifery and Paramedicine, Australian Catholic University, Victoria, Australia; e Public health, Federation University Australia, Victoria, Australia; f Federation University of Australia, Victoria, Australia

**Keywords:** Behavioral change, Cardiovascular diseases, Lifestyle change, Protection Motivation Theory

## Abstract

**Objective:**

To identify and synthesize the primary evidence on the effectiveness of Protection Motivation Theory on and cardiovascular disease and diseases that are risk factors for cardiovascular disease.

**Method:**

An integrative review was conducted using the Whittemore and Knafl method (2005).

**Results:**

Eleven articles met the inclusion and quality assessment criteria. The integration of evidence was abundant in three themes 1) Physical activity 2) Weight and Body Mass Index, and 3) Food consumption and each theme having the same six sub-themes of self-efficacy, response-efficacy, response cost, severity, vulnerability and reward. No studies have addressed all clinical factors and behavioral changes associated with cardiovascular disease.

**Conclusion:**

Due to the limited literature on the effectiveness of Protection Motivation Theory on behavioral changes in patients with cardiovascular diseases, generalizations and practice recommendations are limited. Further research is required to evaluate the effectiveness of this theory in patient outcomes.

## Contribution of the paper

What is already known about the topic?

• Protection Motivation Theory explains how people react to conditions that arouse fear.
• Age differences are considered a factor in provoking fear. Those aged over 50 years, compared to younger people, respond less to messages with fear content, such as perceived vulnerability and severity.

What this paper adds

• In cardiovascular disease, no previous studies have thoroughly investigated the effect of a conceptual theory such as Protection Motivation Theory on the maintenance and continuation of healthy behaviors regarding risk factor modification. Applying this conceptual theory might determine the main factors that affect a patient's desire for effective behavioral change.
• Further research needs to focus on motivational intervention as an essential process for behavioral change and operate clinically to examine if this approach is equally practical and suitable for patients in a clinical environment.
• The impact of Protection Motivation Theory on diseases that are risk factors for cardiovascular disease

## 1 Introduction

Cardiovascular disease is a significant cause of death worldwide, considered the single most common cause of death globally over the last decade (Roth et al., 2017). Cardiovascular disease is a preventable disease, the risk factors of which are controlled by smoking cessation, eating a healthy diet, preventing obesity, addressing insufficient physical activity, and mindfulness (Volpe and Battistoni, 2018). Early risk factor modification may be beneficial for reducing the incidence of hospitalization (Benjamin et al., 2019). Individuals with cardiovascular disease must be aware of the disease and its management. In addition, they need to be more confident about self-management strategies to permanently improve their health condition (Peixoto et al., 2015). Cardiovascular disease progression is delayed for some people by increasing medication adherence and following a healthy lifestyle. People diagnosed with cardiovascular diseases, such as acute coronary syndrome, hypertension, atrial fibrillation, and heart failure, often experience admission and readmission complications that are potentially preventable with suitable education strategies (Peixoto et al., 2015). The positive benefits of secondary prevention programs for cardiovascular disease using multimodal and behavioral interventions include nutritional advice, good physical activity, weight management, smoking cessation, and medication adherence (Whelton et al., 2018).

Patient education is a complex intervention involving various therapeutic areas, including risk factor modifications, behavioral therapy, habit changes, and psychological support to prevent disease causes (Lynggaard, 2019). Several behavioral interventions can be considered in a patient education plan to promote risk factor modifications, improve health outcomes, decrease complications, and reduce hospitalization and readmission (Bitsch et al., 2018). Patient education programs can be delivered in various ways, such as in a classroom as a lecture and group discussion, or individually at the hospital, as a home-visit, and as a telephone follow-up (Barnason et al., 2017). Patient education strategies can be affected by their duration, frequency, maintenance, and reinforcement (Grace et al., 2016). Educational materials for patient education can range from basic printed resources to advanced technology. Factors such as aligning information with patient needs, tailoring content to health literacy and cultural beliefs, and tracking patient progress all play a role in their effectiveness. Healthcare providers can utilize patient health literacy assessments to match material readability, identify the need for additional teaching, and introduce various educational formats (Grace et al., 2016).

Recommendations are practical only when they are accepted by both health professionals and patients. Adherence to advice can be boosted by mutual decision-making between health professionals, patient engagement in all hospitalization processes, and follow-up based on individual values (Arnett et al., 2019). Patient education methods in the cardiovascular field require high patient involvement to promote adherence to cardiac rehabilitation (Lynggaard, 2019). Patient engagement in cardiac rehabilitation programs can improve the quality of life and reduce mortality and morbidity(Bitsch et al., 2018).

An issue of major importance in the cardiovascular field is that many individuals with cardiovascular disease fail to sustain lifestyle improvements (Grace et al., 2016). Considering the effects of a patient education program are vital for improving the methods that help people permanently change their behavior toward a healthier lifestyle, strategies for patient education are established based on validated theories. One of the theories that has been used in different medical fields for risk factor modification is the Protection Motivation Theory (PMT).

Rogers developed the PMT model in 1983 to better understand how and why individuals respond to potential threats to their health and safety. With PMT, individuals cognitively engage in protective behaviors through emotional fear (Rogers and Prentice-Dunn, 1997). PMT has been applied in various studies to encourage people to follow healthy behaviors to prevent diseases. These studies include the use of condoms (Zhang et al., 2015), pap-smear tests (Dehdari et al., 2014), exercise and engagement in suitable physical activity (Mirkarimi et al., 2015), vitamin E and C consumption (Nabizadeh et al., 2018), vaccination decisions (Leder et al., 2015), omega-3 fatty acid consumption (Calder et al., 2011), sun protection behavior (Ch'ng and Glendon, 2014), and complementary and alternative medicine (Kristoffersen et al., 2017). PMT is recommended as an appropriate theoretical framework for developing interventions for various diseases. These include depression, alcohol drinking (Park and Kim, 2017), cervical cancer (Bai et al., 2018), breast cancer (Karmakar et al., 2017), colon cancer (McGowan and Prapavessis, 2010), prostate cancer (Modeste et al., 2018), skin cancer (Taheri et al., 2019), respiratory infection and pandemic flu (Bin et al., 2017; Miller et al., 2012), and type 2 diabetes (Amuta et al., 2016). The only systematic literature review relevant to risk factors in the cardiovascular field was published by Bui et al. (2013), in which PMT was applied to the general population to increase physical activity. However, many studies have focused on only one risk factor in populations without chronic diseases.

The theory's main contribution is its capacity to predict individuals’ intentions to protect themselves after a fear-arousing experience (Boss et al., 2015). Applying this theory persuades patients to follow healthcare professionals’ recommendations. Therefore, the level of intention indicates the usefulness of the attempted persuasion (Boss et al., 2015). The two components of PMT are threat appraisal and coping appraisal for shaping protection intentions. These two processes must occur to engage in an adaptive response (Maddux and Rogers, 1983). First, threat appraisal includes the self-estimated probability of suffering from the disease (perceived vulnerability) and self-estimated seriousness of the disease (perceived severity), which must be more potent than maladaptive behaviors (behaviors that lead the person toward health risks). Second, coping appraisal includes the effectiveness of recommendations to remove or change the threat (response efficacy) and the individual's ability to accomplish the recommended behaviors (self-efficacy), which must outweigh the response costs of implementing protection motivation (Maddux and Rogers, 1983). In 1983, Rogers revised the PMT to create a more comprehensive model. This updated version incorporated adaptive response costs and maladaptive response rewards into the cognitive mediation equation. The inclusion of rewards and costs indicated a significant emphasis on the rationality of decision-making (Maddux and Rogers, 1983).

Fear is another interpersonal construct of PMT provoked in response to a dangerous situation, leading to protective action. Fear plays the role of a stimulus in threat and coping appraisal processes. Fear has been observed to be effective in causing attitude change, which depends on the severity of the threatened event, probability of the event, and efficacy of the recommended response (Maddux and Rogers, 1983). This study aimed to conduct an integrative review of the primary evidence on the effectiveness of PMT on cardiovascular diseases and diseases that are risk factors for cardiovascular disease.

## 2 Methods

Whittemore and Knafl's (2005) integrative review was identified as a suitable method for an inclusive understanding of the phenomenon. This review considered five stages: problem identification, systematic literature search, quality data evaluation with appropriate appraisal tools, analysis and coding, and integrating and synthesizing the data (Whittemore and Knafl, 2005).

### 2.1 Search strategy

The following electronic databases were comprehensively searched from December 2010 to December 2023: CINAHL, EBSCO, MEDLINE, EMBASE, the Cochrane Library, PsycINFO, and Google Scholar with the following keywords: Protection motivation theory, cardiovascular disease, lifestyle modifications, behavioral changes, behavioral intention, behavioral motivation, and patient education. These keywords were combined using ‘OR’ and ‘AND’ to identify the relevant evidence.

Most of the studies provided adequate inclusion and exclusion criteria for the application of PMT. However, two studies (Morowatisharifabad et al., 2018; Park et al., 2011) had more limited descriptive analyses. The inclusion criteria for this study were original articles that examined the effectiveness of PMT on risk factors related to cardiovascular disease in adult participants, studies conducted after 2010, and studies published in peer-reviewed journals.

### 2.2 Study selection

**Fig. 1:**
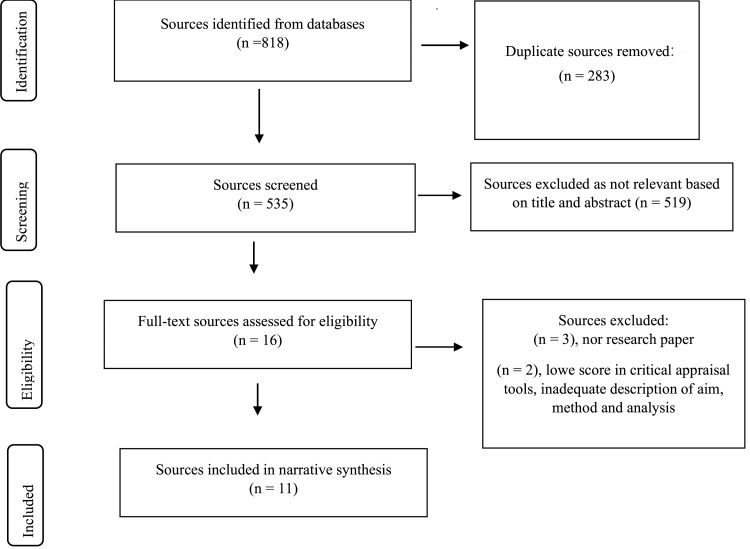
PRISMA flowchart created by Covidence.

All article titles and citation details were retrieved from the databases and exported to the Covidence database for screening. Duplicate references were automatically removed using the Covidence deduplication. Where further duplicates were identified during the screening process, these were manually removed. Titles and abstracts were screened (MG) to exclude articles that did not meet the inclusion criteria. The full text of the article was reviewed if the abstract was not available or if eligibility could not be determined. The reference lists of the articles were reviewed for relevant publications. The full texts of the included articles were screened independently (MG and VP) and disagreements were resolved. A third author (LL) adjudicated where consensus could not be reached. Referring to Fig. 1, the PRISMA flowchart was created by Covidence.

### 2.3 Data extraction and coding

For eligible articles, extracted data included samples, methods, interventions, and outcomes. Data for each study included publication year, country, setting, recruitment process, and design. The sample characteristics included age, sex, and work designation. The research intervention included strategy or program, content, duration, assessment, communication methods, and main findings. Content analysis was used to arrange text that contained a keyword or phrase, arranged into units of meaning, and then allocate a category that may overlap with others. The data were codified and integrated into three finite themes that reflected the answers to review questions. The reliability of the analysis was supported by performing the coding independently by two authors (MG and VP). Any discrepancies were adjudicated by the third author (LL). Each theme involved reviewing the findings of all the research studies. Data comparison and clustering were performed to identify three themes and six subthemes. Subsequently, data integration was applied for the synthesis review (Whittemore and Knafl, 2005).

### 2.4 Search outcomes

Eight hundred and eighteen articles were identified through the database screening, CINAHL 209, EBSCO 130, MEDLINE 356, EMBASE 33, Cochrane Library 12, PsychINFO 76, and Google Scholar 2. Please refer to Fig. 1 for the full search strategy for all databases.

After removal of duplicates, articles remaining were screened for inclusion by title and abstract. After screening the titles, 421 articles were excluded because they had a different research focus (neither cardiovascular nor a cardiovascular disease risk factor) or used other theories or strategies (not PMT). A further 114 articles were eliminated after evaluating the abstracts, leaving 16 potential articles for full-text assessment. Three full-text articles were excluded because they were not research papers, and two articles were excluded because they did not meet the minimum research criteria using critical appraisal tools (Appendix 1).

### 2.5 Quality appraisal process

Eleven articles were subjected to a quality appraisal process to ensure that the minimum research criteria were met. Aspects of the research, including design, recruitment, data collection, ethics, the rigour of the data analysis, results, and the significance of the study to practice, were examined. Eleven articles were included in the review for the analysis and thematic integration of the research findings (see Fig. 1). The data evaluation stage involved using the Joanna Briggs Institute (JBI) Quality Appraisal checklist (2020). Studies that met the inclusion criteria based on title and abstract review were independently in full by two authors to confirm eligibility and to assess methodological quality using the appropriate Joanna Briggs Institute (2020) quality appraisal tools (Buccheri and Sharifi, 2017). Two tools were used, including checklists for analytical cross-sectional studies and randomized controlled trials (The Joanna Briggs Institute, 2020). Since there is no specific tool to evaluate descriptive studies, the checklist for analytical cross-sectional studies was adapted for this purpose (Cooper et al., 2021) (Appendix 2). Quality appraisal criteria were thoroughly discussed, and consensus was reached on which criteria were essential, as well as any modifications and considerations in assessing criteria for each tool (Appendix 3).

### 2.6 Data synthesis

The first author began the initial analysis, and then all articles were reviewed independently by the second author, and the emerging themes were agreed upon. Themes were then developed by involving all authors. Articles were initially categorized based on risk factors for cardiovascular diseases. The three coded themes and six subthemes are listed in Table 1. All primary sources were reviewed to ensure that the defined themes were appropriate following the integration and synthesis of data (Whittemore and Knafl, 2005)

**Table 1:** Themes and subthemes identified from the literature.

References	Morowatisharifabad et al. (2018)	Mirkarimi et al. (2017)	Bassett and Ginis (2011)	Gaston and Prapavessis (2014)	Plotnikoff et al. (2010)	Wong et al. (2016)	Huang (2012)	Redd (2013)	Mirkarimi et al. (2015)	Park et al. (2011)	Zhang and Cooke (2012)
Themes and subthemes	1	2	3	4	5	6	7	8	9	10	11
**Theme 1. Physical activity intention and behavior**											
i) Self-efficacy	√	√		√	√		√				
ii)Response-efficacy	√	√		√	√	√					
iii)Response cost							√				
iv)Severity	√	√	√	√	√	√					
v)Vulnerability				√	√	√	√				
vi)Reward											
**Themes 2. Weight and BMI intention and behavior**											
i)Self-efficacy								√	√		
ii)Response-efficacy								√	√		
iii)Response cost								√	√		
iv)Severity								√	√		
v)Vulnerability								√	√		
vi)Reward									√		
**Theme 3. Healthy food consumption intention and behavior**											
i)Self-efficacy										√	√
ii)Response-efficacy										√	√
iii)Response cost											
iv)Severity										√	√
v)Vulnerability										√	√
vi)Reward											

The PRISMA checklist is an ideal reporting item for the scoping review (Nyanchoka et al., 2019), was used to report the review transparently.

## 3 Results

Eleven articles were included in the integrative literature review (Fig. 1). They are all related to the risk factors for cardiovascular diseases, such as inadequate physical activity, being overweight, and an unhealthy diet. The included studies were conducted in five countries and five regions. Two studies were conducted in Europe, three in the Americas, three in Asia, two in Canada, and one in Australia. The range of sample sizes in the studies varied from 60 to 2311 participants. The participants were male and female, except for three studies with only female participants (Gaston and Prapavessis, 2014; Mirkarimi et al., 2017; Mirkarimi et al., 2015) and their ages ranged from 18 to 65+ years. Two studies used descriptive analysis (Morowatisharifabad et al., 2018; Park et al., 2011), six were RCTs (Gaston and Prapavessis, 2014; Huang, 2012; Mirkarimi et al., 2017; Mirkarimi et al., 2015; Redd, 2013; Zhang and Cooke, 2012), and three used cross-sectional methods (Bassett and Prapavessis, 2011; Plotnikoff and Higginbotham, 2010; Wong et al., 2016). Themes were identified for physical activity, weight, BMI, and diet categories. There were some similarities in the variables associated with behavioral change and intention among the included studies in each category.

The data collection method for predominant protection motivation theory constructs typically involves using surveys or questionnaires to assess individuals' perceived threats and coping appraisals in relation to specific health behaviour. This may include asking participants to rate their perceived severity and susceptibility to a particular threat and their perceived self-efficacy and response efficacy in adopting protective behaviors. Researchers may also utilize qualitative methods such as interviews or focus groups to better understand individuals' motivations and decision-making processes related to health protective behaviors. PMT is concentrated on the prediction of behavioral change and intention in specific conditions, such as patients with type 2 diabetes (Morowatisharifabad et al., 2018; Redd, 2013), type 1 and 2 diabetes (Plotnikoff et al., 2010), spinal cord injury (Bassett and Prapavessis, 2011), and overweight women (Mirkarimi et al., 2017; Mirkarimi et al., 2015). Some studies have investigated the effect of PMT constructs on the prediction of behavioral change and intention in the general population (Zhang and Cooke, 2012) young female college students (Redd, 2013), pregnant women (Gaston and Prapavessis, 2014), university students (Wong et al., 2016), and junior high school students (Huang, 2012). Integrating the findings of the research studies into each theme and subtheme is reported in the following sections.

### 3.1 Physical activity

All studies concluded that there was a positive link between improvements in physical activity and the application of PMT. However, it is also important to consider other factors. As Morowatisharifabad et al. (2018) demonstrated, physical activity remains low among Iranian women with type 2 diabetes despite repeated recommendations. Morowatisharifabad et al. (2018) noted that social, cultural, and demographic factors, such as level of education and income, play a crucial role in maintaining a low level of physical activity. However, it is also necessary to implement strategies to increase physical activity, Table 2.

**Table 2:** Relationship of PMT actions for improving physical activity in patients with different risk factors for CVD.

**Article number**	**Title and Authors**	**Setting**	**Methods**	**Sample characteristics**	**Findings**
1.	“The Predictive Effects of Protection Motivation Theory on Intention and Behaviour of Physical Activity in Patients with Type 2 Diabetes.” Morowatisharifabad et al. (2018)	Iran	Descriptive-analytical study with International Physical Activity Questionnaire-Short Form (IPAQ-SF) Self-design PMT questionnaire	N = 250 Men: 190 Women: 60	The PMT explained the highest correlation between self-efficacy and intention to do physical activity (r = 0.716) and the lowest correlation with perceived severity (r = 0.171). In Model 1, with the six PMT constructs, self-efficacy and perceived cost had a higher prediction of behavior (p < 0.001). In Model 2, with two PMT processes, coping appraisal predicted physical activity intention (p < 0.001) and Model 3 after controlling confounding factors. No change in the ability to predict physical activity intention has been observed.
2.	“Modifying attitude and intention toward regular physical activity using protection motivation theory.” Mirkarimi et al. (2017)	Iran	RCT two months and six months follow-up Post-intervention	N=60 Women BMI = 25–29.9 (overweight) or 30–35 (obese), being literate ability to exercise	Perceived response efficacy (p < 0.001), severity (p < 0.014), and self-efficacy (p < 0.043) predicted intention at two months follow-up. Perceived severity (p < 0.020) predicted intention at six months of follow-up. Self-efficacy (p < 0.009) was only predictor for changing attitude to do physical activity at two months follow-up and vulnerability (p < 0.042) and self- efficacy (p < 0.001) were at six months follow-up.
3.	“Risky business: The effects of an individualized health information intervention on health risk perceptions and leisure-time physical activity among people with spinal cord injury.” Bassett and Ginis (2011)	Canada	Cross- sectional study	N = 62 Men: 52 Women: 10 Mean age: 43 years	Perceived risk of cardiovascular disease was correlated with decreased leisure-time physical activity (p < 0.10). Baseline perceived risk of obesity and diabetes was a positive predictor of increased leisure-time physical activity (p < 0.05)
4.	“Using a combined protection motivation theory and health action process approach intervention to promote exercise during pregnancy.” Gaston and Prapavessis (2014)	Canada	RCT with two different intervention plans with baseline (time 1), immediate (time 2), and after four weeks (time 3) follow-up	N= 60 Pregnant women	Both action plan and combined planning groups had significantly higher action planning than the PMT group. The combined planning group also had significantly higher coping planning four weeks post-intervention (p < 0.001). It means the combination of PMT with a Health Action Process Approach is more beneficial (p < 0.001).
5.	“Protection motivation theory and the prediction of physical activity among adults with type 1 or type 2 diabetes in a large population sample.” Plotnikoff et al. (2010)	Australia	Cross-sectional one-week pre intervention (time 1), one-week post intervention (time 2), and six months follow-up (time 3)	N =2311 (697 with T1D and 1614 with T2D) T1D: Men: 46% Women: 54% T2D: Men: 51% Women: 49%	Self-efficacy of coping appraisal was a significant predictor of intention in both diabetic groups (β = 0.64-0.68, p < 0.01). The severity of threat appraisal was an essential variable to intention in T2D only (β = 0.06, p < 0.6)
6.	“The utility of a protection motivation theory framework for understanding sedentary behaviour.” Wong et al. (2016)	Canada	Cross-sectional study	N= 787 University students Aged: 18-35 years	Significant variables that contributed to goal intention were: Response efficacy (β = −.16; p < 0.01) and self-efficacy (β = −.21; p < 0.05) of coping appraisals
7.	“Effects of motivational and volitional interventions on adolescents’ physical activity behavior.” Huang (2012)	USA	RCT with three groups control, motivational group, and volitional group. The week prior to intervention, start point (time 1), in two weeks (time 2), in four weeks (time 3)	N=330 Boys: 163 Girls: 167 Junior high school students	Self-efficacy (p < 0.01), response efficacy (p < 0.01), and response costs (p < 0.01) of coping appraisal were significantly correlated with physical activity behavior and intention. Perceived vulnerability (p < 0.01) of threat appraisal was correlated with behavior and intention (p < 0.01, p < 0.05, respectively.

There was a meaningful relationship between physical activity intention and PMT constructs. Self-efficacy was one of the most important predictors of predicting behavior for physical activity in some studies (Bui et al., 2013; Gaston and Prapavessis, 2014; Huang, 2012; Mirkarimi et al., 2017; Plotnikoff and Higginbotham, 2010; Wong et al., 2016). An increased perceived severity of immobility can lead individuals to more physical activity motivation if they believe that the response is effective (Bui et al., 2013). Response efficacy has also been a significant predictor of physical activity in other studies (Huang, 2012; Mirkarimi et al., 2017; Morowatisharifabad et al., 2018; Wong et al., 2016). Self-efficacy is the strongest predictor of intention, where there was a longer-term follow-up of more than three months. This is explained by the fact that attitude change requires more time than other constructs (Mirkarimi et al., 2017). Mirkarimi et al. (2017), in their RCT, compared the study's findings with other studies that had applied motivational intervention combined with volitional intervention. They found that while motivational interventions aim to increase motivation and willingness to change behavior, volitional interventions focus on planning and executing the necessary actions to achieve the desired behavior change. Therefore, Mirkarimi et al. (2017) highlighted that a PMT-centered intervention alone was not enough to bring about behavior modification and emphasized the need for action and coping planning. However, Mirkarimi et al. (2017) also highlighted that the differences in results between studies could be attributed to the different sample populations involved.

Morowatisharifabad et al. (2018) demonstrated no significant correlation between perceived severity and physical activity in patients with diabetes. This finding was inconsistent with the results of Plotnikoff et al. (2010), in which severity was significantly correlated with intention in patients with type 2 diabetes (p < 0.001). Conversely, the relationship between intention and physical activity was not significantly stronger in patients with type 1 diabetes because this group of patients had lower perceived vulnerability, higher self-efficacy, and more baseline physical activity. Therefore, depending on different etiologies and habits, individuals can have various intention modification levels, and interventions are required to increase intention, focusing more on the severity of issues in type 2 diabetes. This means that patients who believe that their disease is severe have a higher intention to be active (Plotnikoff et al., 2010).

Regarding vulnerability, Huang (2012) stated that among threat appraisal constructs, only vulnerability contributed significantly to the plan to modify physical activity intention (p < 0.01) and behavior (p < 0.05). Wong et al. (2016) found that perceived vulnerability had a lower score because participants had a defensive denial of the threat, which blunted its psychological impacts.

In a study performed by Huang (2012), perceived cost had a significant influence on enhancing physical intention and behavior (p < 0.01); however, the construct of coping appraisal had a contrary relationship in predicting the intention to engage in physical activity in another study (Morowatisharifabad et al., 2018). This might be due to differences in health conditions. For instance, people with diabetes may not have significant external and internal rewards for not engaging in physical activity. In contrast, perceived costs such as time spent, expense, tiredness, and weakness can more effectively limit physical activity intention. Therefore, physical activity motivation can succeed by adjusting the perceived costs.

Most studies have found that coping appraisal predicts motivation more than threat appraisal (Gaston and Prapavessis, 2014; Plotnikoff et al., 2010; Redd, 2013; Wong et al., 2016). This outcome aligns with that of a systematic review of the application of PMT to physical activity in the general population (Bui et al., 2013). Bui et al. (2013) stated that coping appraisal could facilitate physical activity intentions in specific populations with an increased risk of chronic health conditions and inactive lifestyles. Twenty studies were reviewed with categories of prediction, stage discrimination, experimental manipulation, and intervention. The results indicated that self-efficacy was the most effective variable for predicting and promoting physical activity. In addition, PMT is for conditions that arouse fear. Participants who are not at risk for chronic health conditions may benefit less, and age differences are considered a factor in provoking fear (Bui et al., 2013). Morowatisharifabad et al. (2018) included participants aged over 50 years. They indicated that older people, compared to younger people, responded less to messages with fear content, such as perceived vulnerability and severity.

Bassett and Ginis (2011) stated that health professionals should provide individual health risk information to increase awareness of inactivity-related disease risks. Risk information stimulates fear and, consequently, may influence perceived severity and vulnerability more than others. Zhang and Cooke (2012) also reported that, although a motivational intervention based on PMT constructs had significant positive effects on intention and behavior, further research will investigate the best way of manipulating PMT constructs with a factual health education intervention to increase intention and change behavior.

### 3.2 Weight and BMI

Two articles in the weight and BMI category (Table 3) aimed to decrease weight and BMI among young adult female college students with a diabetic history (Redd, 2013) and overweight women (Mirkarimi et al., 2015). Mirkarimi et al. (2015) performed an RCT with a motivational interview and intention intervention for an experimental group to explore the effect of PMT on weight loss intention. A significant relationship was observed between weight loss intention and PMT construction. Self-efficacy is one of the essential constituents in predicting behavior for weight loss (p = 0.001), which is similar to a study by Redd (2013) (p < 0.01). Perceived response efficacy (p = 0.001) also significantly predicted participants’ intentions (Mirkarimi et al., 2015). The perceived reward of threat appraisal was able to predict the participant's intentions (p = 0.022).

**Table 3:** Relationship of PMT actions for decreasing weight and BMI in patients with different health conditions and the general population.

**Article number**	**Title and Authors**	**Setting**	**Methods**	**Sample characteristics**	**Finding**
8.	“Using the protection motivation theory to examine the effects of obesity fear arousal on the physical activity of young adult female college students.” Redd (2013)	USA	RCT with two groups of control and intervention groups. Baseline (time 1), in two weeks (time 2), in 4 weeks (time 3) post-intervention	N = 250 Men: 60 Women: 190 Aged: 28-65 years Diabetic history BMI: N = 61 > 30 N = 116, 25-29.9	Two constructs of coping appraisal, self-efficacy, and response costs had the highest predictability of the intention of physical activity p < 0.01). Response efficacy was moderately high at times 2 and 3 (p < 0.01).
9.	“Effect of Motivational Interviewing on a Weight Loss Program Based on the Protection Motivation Theory.” Mirkarimi et al. (2015)	Iran	RCT with three groups of the standard weight control program, motivational interviewing, and motivational intervention plus intention intervention. Baseline and two months follow-up	N= 150 Women BMI 25-29.9 (overweight) and 30- 35 (obese)	Self-efficacy (p = 0.001), response efficacy (p = 0.001), coping appraisal, and rewards (p = 0.022) of threat appraisal were significant predictors for intention.

Redd (2013) used PMT to examine the effects of obesity fear arousal on physical activity among overweight female students. This study demonstrated that most students had positive self-efficacy regarding their ability to perform physical activity and positive response efficacy regarding their ability to undertake physical activity to decrease the risk of being overweight. Overall, high self-efficacy and response efficacy perceptions decreased vulnerability (p < 0.06) in all groups. Redd (2013) suggested that the two most important predictive factors are low-risk participants and past performance trends.

Redd (2013) suggested that the implementation of intention interventions based on PMT constructs successfully changed behavior in obesity. The duration and frequency of the programs can influence the effectiveness of patient education strategies within PMT interventions. Longer-term interventions can offer more comprehensive education and behavior change opportunities, while frequent sessions can reinforce patient education, aiding in information retention and skill development. Sustaining the effects of patient education strategies over time is crucial, and ongoing support and follow-up can enhance their long-term impact. Therefore, it is essential to understand and address the different elements of patient education strategies within the context of PMT to optimize their effectiveness in promoting positive health outcomes. Employing PMT altered constructs like perceived sensitivity, perceived severity, knowledge, and behavior motivation among different studies (Huang, 2012; Mirkarimi et al., 2017; Mirkarimi et al., 2015; Redd, 2013). This suggests that educational interventions utilizing PMT could be beneficial for addressing other diseases as well. Identifying preventive measures, control strategies for chronic disease, and understanding the factors influencing individuals' adoption of preventive behaviors are vital in addressing these issues. However, further research is needed to examine the effects of patient education interventions on the subsequent cognition of PMT constructs. Table 3 demonstrated the relationship between PMT interventions aimed at reducing weight and BMI in individuals with diverse health conditions.

### 3.3 Food consumption

The food consumption category included two articles that applied interventions to promote a healthy diet among the general population (Park et al., 2011; Zhang and Cooke, 2012). Zhang and Cooke (2012) argued that motivational interventions created significant changes in response efficacy when following a healthy diet (p < 0.001) and regular physical activity (p < 0.001). Participants who received the motivational intervention had a higher consumption of fruits and vegetables.

These results are consistent with those of Park et al. (2011), who found that PMT successfully influenced variables related to behavioral change and intentions and produced essential changes for the coping appraisal process. The interventions highlighted important information about reducing risks by incorporating a healthy diet and exercise into routine daily life. Park et al. (2011) adapted the PMT model to measure adult consumers’ behavior and intentions regarding the consumption of functional foods (functional foods are enriched foods that contain essential nutrients, including fruits, vegetables, nuts, and seeds). Threat appraisal (severity and vulnerability) was not a significant predictor; this outcome differs from that of Zhang and Cooke (2012). Non-significant outcomes of threat appraisal might be attributed to the nature of the participants, who were between 18 and 34 years of age without any health conditions, which might lead to lower intention to change behavior. Self-efficacy was the most significant predictor of intention (p < 0.001) and behavior (p < 0.05). Response efficacy was a significant predictor of intention to eat a healthy diet (p < 0.001) (Park et al., 2011). Overall, these two studies emphasized the importance of coping appraisal. Response efficacy plays a role in influencing the actual improvement in consuming a healthy diet indirectly through intention and an individual's belief about consuming a healthy diet (Zhang and Cooke, 2012). In contrast, self-efficacy predicts an individual's ability to consume a healthy diet (Park et al., 2011). Table 4 demonstrated the relationship between PMT interventions aimed at increasing healthy dietary behaviour in patients with diverse health conditions and the general population.

**Table 4:** The relationship of PMT actions to increase healthy dietary behaviour in patients with different health conditions and the general population.

**Article Number**	**Title and Authors**	**Setting**	**Methods**	**Sample characteristics**	**Finding**
10.	“The Use of the Modified Protection Motivation Theory to Explore Adult Consumers’ Functional Foods Consumption Behavior.” Park et al. (2011)	USA	Descriptive - analytical study with the online survey questionnaire	N = 465 Men: 25.1% Women: 74.9%	Self-efficacy of coping appraisal on intention (p < 0.001) and behavior (p < 0.05) is the best predictor regarding intention and behavior. Response efficacy was a significant predictor of intention (p < 0.001), not behavior.
11.	“Using a combined motivational and volitional intervention to promote exercise and healthy dietary behaviour among undergraduates.” Zhang and Cooke (2012)	UK	RCT with three experimental groups of motivational intervention, volitional intervention, and a combination of both interventions. Baseline, two and four weeks follow-up	N = 84 Men: 43 Women: 41 Aged 18- 24 years No history of diabetes	Response efficacy of coping appraisal is the strongest predictor of eating a healthy diet (p < 0.001) and regular exercise (p < 0.001)

## 4 Discussion and conclusion

Self-efficacy is an important promoting factor in motivational and cognitive processes to increase the probability of selecting adaptive responses and protective actions (Henson et al., 2010). Self-efficacy and response efficacy are two constructs that are linearly related to the influential indicators of obtaining intention.

All the studies included measures of severity, vulnerability, response efficacy, and self-efficacy in physical activity. Response cost was considered in two studies (Huang, 2012; Morowatisharifabad et al., 2018). All studies in the physical activity category measured both physical activity intention and behavior except for one that measured intention (Bassett and Prapavessis, 2011). The intention in all studies in the physical activity category was a precursor to the behavior, increasing the likelihood of engagement in a particular behavior. Behavioral measurements for physical activity included self-reported frequency of vigorous or moderate physical activity sessions, calculated calorie expenditure, total time, and the number of days per week spent in physical activity. In their study, Morowatisharifabad et al. (2018) converted behavioral measures to metabolic rate per minute (MetS).

Overall, the PMT variables were significantly associated with physical activity intention and behavior. Self-efficacy appeared to be the strongest predictor in all studies in the physical activity category (Gaston and Prapavessis, 2014; Huang, 2012; Mirkarimi et al., 2017; Morowatisharifabad et al., 2018; Plotnikoff and Higginbotham, 2010; Wong et al., 2016). The response efficacy of coping appraisal was also one of the strongest predictors of intention and behavioral change in three studies (Huang, 2012; Mirkarimi et al., 2017; Wong et al., 2016). Two studies significantly correlated The response cost with intention and behavioral change (Huang, 2012; Morowatisharifabad et al., 2018).

Regarding threat appraisal, Bassett and Prapavessis (2011) demonstrated that the perceived severity of the risk of diabetes and cardiovascular disease is a positive predictor of leisure-time physical activity (p < 0.05, p < 0.10). Plotnikoff et al. (2010) stated that higher perceived severity was linked only to higher intention in the T2D group (β = 0.06). Following this study, the risk factors for CVD are of particular interest, consistent with the study highlighted that complementary and alternative use might be motivated by a protective response to perceptions of risk for developing coronary heart diseases (Kristoffersen et al., 2017). All four of the PMT components were associated with complementary and alternative medicines used in the risk group in this study, which suggested that people use the medicines to respond to a health threat, prevent disease, and maintain health (Kristoffersen et al., 2017). Mirkarimi et al. (2017) found that severity was the strongest predictor of performing physical activity (p = 0.20). Conversely, perceived severity was the lowest predictor of intention and behavioral change (r = 0.0741) (Morowatisharifabad et al., 2018). Vulnerability was significantly correlated with behavioral change (r = -0.23, p < 0.01) and intention (r = -0.14, p < 0.05) to perform physical activity (Huang, 2012).

In the weight and BMI category (Table 3), regarding coping appraisal, self-efficacy (p < 0.01) and response cost (p < 0.01) had the highest relationship with intention and behavioral change (Redd, 2013), and all three components of coping appraisal self-efficacy (p = 0.001), response efficacy (p = 0.001) and response cost (p = 0.014) were the highest predictors for intention and behavioral change (Mirkarimi et al., 2015). Regarding threat appraisal, vulnerability was moderately high at the second and third follow-ups (p < 0.01) (Redd, 2013). By contrast, Mirkarimi et al. (2015) demonstrated that severity (p = 0.001), vulnerability (p = 0.001), and rewards (p = 0.004) were the strongest predictors of intention and behavioral change. The only study in this literature review that considered rewards as a threat appraisal construct was Mirkarimi et al. (2015), which is supported by the study demonstrated that threat appraisal toward non-communicable diseases such as cardiovascular disease and hypertension was significantly higher than among healthy participants (Chamroonsawasdi et al., 2017).

The investigation of healthy dietary behavior (Table 4) indicated that self-efficacy of coping appraisal in intention (p < 0.001) and behavior (p < 0.5) was the strongest predictor. In contrast, response efficacy was a significant predictor of intention (p < 0.001) (Park et al., 2011). Zhang and Cooke (2012) also revealed that coping appraisal was the strongest predictor of eating a healthy diet (p < 0.001) and regular exercise (p < 0.001). None of the studies measured threat appraisal constructs.

The findings of this review support the application of PMT in promoting physical activity, decreasing weight and BMI, and eating a healthy diet. There were generally positive effects of the interventions on intentions and behavior. Patient education strategies in the cardiovascular field have been applied to enhance physical activity, eat a healthier diet, and stop smoking. However, any associated improvement in reducing lipid profiles, medication adherence, and mindfulness is ambiguous in this field.

The coping appraisal construct of self-efficacy was the most influential variable in predicting healthy behavior and promoting intention. However, extensive long-term studies need to consider interventions to identify barriers to increasing adherence to behavioral change plans. Investigating the risk factors affecting behavioral changes among patients in clinical settings with chronic conditions is necessary. Motivational interventions can decrease the risk factors among these patients (Huang, 2012; Mirkarimi et al., 2015; Zhang and Cooke, 2012).

The PMT variables were found to have a significant link with healthy intentions and behaviors. While PMT indicates promise for predicting the adoption of healthy behaviors, few studies have investigated the effectiveness of PMT constructs in predicting physical activity intentions and behavior. In addition, no study has been found that evaluated the effectiveness of PMT constructs as a strategy for post-discharge patient education in high-risk groups such as patients with diabetes, chronic disease, or cardiovascular disease. Further research on the operationalization and testing of PMT interventions in an experimental environment is needed, which might assist in finding a tailored strategy for interventions in individuals with chronic diseases. Despite highlighting the significance of PMT in three domains, this integrative review has some limitations. Biases might be presented to the methodological restrictions of the review to English-written studies and key words used. Secondly, quality limitations of the included studies, such as volenteer samples of women and men withouth specific disease, volenteer students, or only speficially on women with obesity and women with pregnancy. However, all of studies included in the current review were of moderated higher scientific and adequate finding validity. Thirdly, inconsistent measures of physical activity and sedentary time using a self-report method could limit the comparability of the findings. Fourthly, some of the studies only measured intentions, not behavior. Therefore, it is unidentified whether intention would lead to behavior modification. Finally, behavior change is a highly complex concept. However, many of these complexities can be addressed by knowing the complexities and carefully considering the individualized application of PMT. PMT provides an excellent starting model to enhance and further develop our understanding of the intrinsic and extrinsic factors that encourage or discourage the usage of protective measures in response to healthy behaviors, how individuals assess the protective measures, and whether or not to utilize them. Rigorous interventions with PMT applications should be considered in the battle for chronic disease prevention and health enhancement. Considering the imperative need to enforce protective measures against chronic disease and strictly adhere to health protocols within the healthcare profession, it is absolutely crucial to thoroughly examine the attitudes and beliefs surrounding the rigorous implementation of these behaviors among healthcare workers. This analysis will unequivocally inform the development and execution of appropriate educational interventions aimed at fostering a culture of adherence and promoting these protective behaviors.

### 4.1 Practice implications

Several strategies have been implemented to expand motivation for lifestyle modifications. PMT usage in different studies has motivated patients to improve self-management, knowledge of the disease, understanding of their need to manage their illness and living with the chronic condition, recognition of changes in condition, adaptation of recommended lifestyle changes, and self-monitoring of their risk factors (Clubb and Hinkle, 2015). This integrative review provides evidence in encourage of expanding the PMT and using it to facilitate behavioral change in effectively engaging with patients with chronic diseases. This approach appears promising and merits further investigation. The theory's comprehensive nature, coupled with its practicality, suggests its potential acceptance and its capacity to improve procedures and outcomes in complex conditions, which often have long-term adverse effects. Long-term patient education interventions by multidisciplinary teams need to specify the focal behaviors and patients’ needs to manage chronic conditions. In cardiovascular disease, no previous studies have thoroughly investigated the effect of a conceptual theory such as PMT on maintaining and continuing healthy behaviors regarding risk factor modification. Applying this conceptual theory might determine the main factors that affect a patient's desire for effective behavioral change. Exploring the long-term impact of PMT-based interventions on sustained behavior change, especially in the context of chronic disease management, is vital for achieving long-term behavior change.

## Ethics approval and consent to participate

This study is an integrative review of published literature. Ethical approval and consent to participate were not applicable in this review.

## Consent for publication

Not applicable.

## CRediT authorship contribution statement

**Maryam Ghasemiardekani:** Writing – original draft, Software, Project administration, Methodology, Investigation, Formal analysis, Conceptualization. **Virginia Plummer:** Validation, Writing – review & editing. **Louisa Lam:** Supervision, Validation, Writing – review & editing. **Biswajit Banik:** Writing – review & editing, Supervision. **Wendy Cross:** Supervision, Writing – review & editing.

## Declaration of competing interest

The authors declare that they have no known competing financial interests or personal relationships that could have appeared to influence the work reported in this paper.

## Figures and Tables

**Table:** 

Search strategy	search terms	
**A Population**	#1 "cardiovascular diseases" OR "myocardial infarction" OR "heart diseases" OR "heart failure" OR "cardiac disease*" #2 "chronic disease” OR " OR "noncommunicable diseases" OR "noncommunicable disease*" OR "NCD" OR "NCDs" #3 “diabetes mellitus, type 2” OR DMII OR DM2 OR “type 2 diabetes” OR “diabetes mellitus type 2” OR NIDDM OR “impaired glucose tolerance” #4 "respiratory disease" OR "lung diseases" OR "respiratory illness" OR "respiratory condition" OR "pulmonary disease" OR "pulmonary disease, chronic obstructive” OR "pulmonary condition" OR "COPD" OR "asthma" #5 "musculoskeletal disorder" OR "musculoskeletal diseases" OR "musculoskeletal condition" OR "back pain" OR arthrosis" OR "osteoporosis" OR "osteoporosis" OR " rheumatoid arthritis" #6 #1 OR #2 OR #3 OR #4 OR #5	
**B Outcome**	AND # 7 “self-management” OR “patient self-management” OR “self-care” OR “patient self-care” OR “self-medication” OR self-administration” OR “blood glucose self-monitoring” OR “self-monitor” OR “self-efficacy” #8 “patient-education” OR “behaviour therapy” OR “patient-focused” OR "lifestyle “ OR "lifestyle modification” OR "lifestyle change" OR “lifestyle adjustments” OR "health behaviour" OR “ behavioural change” OR “ behavioural adjustment” OR "patient preference” OR "patient participation” OR "disease management" OR "disease progression" OR "hospital admission" OR "patient readmission" OR “ intention to behavioural change or modification” #9 #7 OR #8	
**C Perception**	AND #10” healthcare professional” OR “healthcare providers” OR “healthcare workers” OR “clinicians” OR “ clinical consultants” #11 "factor “OR "constraint" OR "cause" OR "barrier" OR "facilitator" OR "enabler" OR "predictor” OR "outcome" OR "knowledge" OR "attitude" OR "perception" #12 #10 OR #11	
**D Intervention**	AND **#**14 “protection motivation theory” OR “PMT” OR “perceived severity” OR “perceived vulnerability” OR “reward” OR “threat appraisal” OR “coping appraisal” OR “response efficacy” OR “behavioural change model” OR” behavioural change theory” OR “motivational intervention” OR “motivational interview” OR “ long-term altering habits intervention” OR “long-term behavioural change intervention” OR “behavioural change strategies” OR “ behavioural change techniques” OR “theories of behavioural change” OR “ behavioural change components” OR “behavioural change variables” OR protective behaviour” OR “cognitive changes” OR behavioural theoretical framework” # 15 “behavioural change stages “OR “behavioural changes example in health” OR “person-centre approach for behavioural change” OR “behavioural change communication” OR “integrated behavioural change model” Behavioural change toward health promotion” OR “behavioural change practical guide” #16 #14 OR #15	
**Database**	**Search**	**Result: number of hits**
#17 MEDLINE	Search #7 And #10 AND #13 And #16	356
#18 CINAHL	Search #7 And #10 AND #13 And #16	209
#19 EMBASE	Search #7 And #10 AND #13 And #16	33
#20 Google Scholar	Search 14 AND 15	2
#21 EBSCO	Search #7 And #10 AND #13 And #16	130
#22 Cochrane Library	Search #7 And #10 AND #13 And #16	12
#23PshychiNFO	Search #7 And #10 AND #13 And #16	76

**Table:** 

	Yes	No	Unclear	Not applicable
1. Were the criteria for inclusion in the sample clearly defined?	□	□	□	□
2. Were the study subjects and the setting described in detail?	□	□	□	□
3. Was the exposure measured in a valid and reliable way?	□	□	□	□
4. Were objective, standard criteria used for measurement of the condition?	□	□	□	□
5. Were confounding factors identified?	□	□	□	□
6. Were strategies to deal with confounding factors stated?	□	□	□	□
7. Were the outcomes measured in a valid and reliable way?	□	□	□	□
8. Was appropriate statistical analysis used?	□	□	□	□

**Table:** 

	Yes	No	Unclear	NA
Was true randomization used for assignment of participants to treatment groups?	□	□	□	□
Was allocation to treatment groups concealed?	□	□	□	□
Were treatment groups similar at the baseline?	□	□	□	□
Were participants blind to treatment assignment?	□	□	□	□
Were those delivering treatment blind to treatment assignment?	□	□	□	□
Were outcomes assessors blind to treatment assignment?	□	□	□	□
Were treatment groups treated identically other than the intervention of interest?	□	□	□	□
Was follow up complete and if not, were differences between groups in terms of their follow up adequately described and analyzed?	□	□	□	□
Were participants analyzed in the groups to which they were randomized?	□	□	□	□
Were outcomes measured in the same way for treatment groups?	□	□	□	□
Were outcomes measured in a reliable way?	□	□	□	□
Was appropriate statistical analysis used?	□	□	□	□
Was the trial design appropriate, and any deviations from the standard RCT design (individual randomization, parallel groups) accounted for in the conduct and analysis of the trial?	□	□	□	□

**Table:** 

JBI Critical Appraisal Checklist for Descriptive Analysis
Author(s) Q1 Q2 Q3 Q4 Q5 Q6 Q7 Q8 Total Risk^b^ Park et al. (2011) × × ✓ ✓ ✓ ✓ ✓ ✓ 75% Low Morowatisharifabad et al. (2018) ✓ ✓ × ✓ ✓ ✓ ✓ ✓ 87.5% Low
JBI Critical Appraisal Checklist for Analytical Cross-sectional Studies
Author(s) Q1 Q2 Q3 Q4 Q5 Q6 Q7 Q8 Total Risk^b^ Bassett and Ginis (2011) ✓ ✓ ✓ ✓ × × ✓ ✓ 75% Low Wong et al. (2016) ✓ ✓ ✓ ✓ × ✓ ✓ ✓ 87.5% Low Plotnikoff and Higginbotham ✓ ✓ ✓ ✓ × ✓ ✓ ✓ 87.5% Low
JBI Critical Appraisal Checklist for Randomized Controlled Trials
Author(s) Q1 Q2 Q3 Q4 Q5 Q6 Q7 Q8 Q9 Q10 Q11 Q12 Q13 Total Risk^b^ Redd (2013) ✓ ✓ ✓ ? ✓ ✓ ? ✓ ✓ ✓ ✓ ✓ ✓ 85% Moderate Zhang and Cooke (2012) ✓ ✓ ✓ ? ✓ ? ✓ ✓ ✓ ✓ ✓ ✓ ✓ 85% Moderate Gaston and Prapavessis (2014) ✓ ✓ ? ✓ ✓ ✓ ? ✓ ✓ ✓ ✓ ✓ ✓ 85% Moderate Huang (2012) ✓ ✓ ✓ ? ? ? ✓ ✓ ✓ ✓ ✓ ✓ ✓ 77 % Moderate Mirkarimi et al. (2017) ✓ ✓ ✓ ✓ ? ? × ✓ ✓ ✓ ✓ ✓ ✓ 77 % Moderate Mirkarimi et al. (2015) ✓ ✓ ✓ ✓ ? ? × ✓ ✓ ✓ ✓ ✓ ✓ 77 % Moderate

## Appendix 2 Quality Analysis using Joanna Briggs Institute Critical Appraisal Tools

**JBI CRITICAL APPRAISAL CHECKLIST FOR ANALYTICAL CROSS SECTIONAL STUDIES**

Reviewer______________________________________ Date_______________________________

Author_______________________________________ Year_________ Record Number_________

Overall appraisal: Include □ Exclude □ Seek further info Comments (Including reason for exclusion)

**JBI CRITICAL APPRAISAL CHECKLIST FOR RANDOMIZED CONTROLLED TRIALS**

Reviewer______________________________________ Date_______________________________

Author_______________________________________ Year_________ Record Number_________

Overall appraisal: Include □ Exclude □ Seek further info Comments (Including reason for exclusion)

## Appendix 3 Quality Analysis using Joanna Briggs Institute Critical Appraisal Outcomes

Note: The risk of bias was ranked as high when the study reached up to 49% of “yes” scores, moderate when the study reached from 50 to 69% of “yes” scores, and low when the study reached more than 70% of “yes” scores. ‘✓’ indicates yes, ‘✕’ indicates no and ‘?’ indicates unclear.

## References

[bib0001] Amuta A.O., Jacobs W., Barry A.E., Popoola O.A., Crosslin K. (2016). Gender Differences in Type 2 Diabetes Risk Perception, Attitude, and Protective Health Behaviors: A Study of Overweight and Obese College Students. Am. J. Health Educ..

[bib0002] Arnett D.K., Blumenthal R.S., Albert M.A., Buroker A.B., Goldberger Z.D., Hahn E.J., Himmelfarb C.D., Khera A., Lloyd-Jones D., McEvoy J.W., Michos E.D., Miedema M.D., Munoz D., Smith S.C., Jr, Virani S.S., Williams K.A., Sr, Yeboah J., Ziaeian B (2019). 2019 ACC/AHA Guideline on the Primary Prevention of Cardiovascular Disease: Executive Summary: A Report of the American College of Cardiology/American Heart Association Task Force on Clinical Practice Guidelines. J. Am. Coll. Cardiol..

[bib0003] Bai Y., Liu Q., Chen X., Gao Y., Gong H., Tan X., Zhang M., Tuo J., Zhang Y., Xiang Q., Deng F., Liu G. (2018). Protection motivation theory in predicting intention to receive cervical cancer screening in rural Chinese women. Psychooncology..

[bib0004] Barnason S., White-Williams C., Rossi L.P., Centeno M., Crabbe D.L., Lee K.S., McCabe N., Nauser J., Schulz P., Stamp K., Wood K., American Heart Association Council on, C., Stroke, N., Council on Cardiovascular Disease in the, Y., Council on Clinical, C., & Stroke,C (2017). Evidence for Therapeutic Patient Education Interventions to Promote Cardiovascular Patient Self-Management: A Scientific Statement for Healthcare Professionals From the American Heart Association. Circulation: Cardiovascular Quality and Outcomes.

[bib0005] Bassett S.F., Prapavessis H. (2011). A test of an adherence-enhancing adjunct to physiotherapy steeped in the protection motivation theory. Physiotherapy Theory & Practice.

[bib0006] Benjamin E.J., Muntner P., Alonso A., Bittencourt M.S., Callaway C.W., Carson A.P., Chamberlain A.M., Chang A.R., Cheng S., Das S.R., Delling F.N., Djousse L., Elkind M.S.V., Ferguson J.F., Fornage M., Jordan L.C., Khan S.S., Kissela B.M., Knutson K.L., Kwan T.W., Lackland D.T., Lewis T.T., Lichtman J.H., Longenecker C.T., Loop M.S., Lutsey P.L., Martin S.S., Matsushita K., Moran A.E., Mussolino M.E., O'Flaherty M., Pandey A., Perak A.M., Rosamond W.D., Roth G.A., Sampson U.K.A., Satou G.M., Schroeder E.B., Shah S.H., Spartano N.L., Stokes A., Tirschwell D.L., Tsao C.W., Turakhia M.P., VanWagner L.B., Wilkins J.T., Wong S.S., Virani S.S., American Heart Association Council on, E., Prevention Statistics, C., & Stroke Statistics, S (2019). Heart Disease and Stroke Statistics-2019 Update: A Report From the American Heart Association. Circulation.

[bib0007] Bin C., Qiuyan L., Wendy Wing Tak L., Zong Ping L., Fielding R., Cui B., Liao Q., Lam W.W.T., Liu Z.P (2017). Avian influenza A/H7N9 risk perception, information trust and adoption of protective behaviours among poultry farmers in Jiangsu Province, China. BMC. Public Health.

[bib0008] Bitsch B.L., Nielsen C.V., Stapelfeldt C.M., Lynggaard V. (2018). Effect of the patient education - Learning and Coping strategies - in cardiac rehabilitation on return to work at one year: a randomised controlled trial show (LC-REHAB). BMC. Cardiovasc. Disord..

[bib0009] Boss S.R., Galletta D.F., Lowry P.B., Moody G.D., Polak P. (2015). What do users have to fear? Using fear appeals to engender threats and fear that motivate protective behaviors in users. MIS Quarterly.

[bib0010] Buccheri R.K., Sharifi C. (2017). Critical Appraisal Tools and Reporting Guidelines for Evidence-Based Practice. Worldviews. Evid. Based. Nurs..

[bib0011] Bui L., Mullan B., McCaffery K. (2013). Protection motivation theory and physical activity in the general population: a systematic literature review. Psychol. Health Med..

[bib0012] Calder S.C., Davidson G.R., Ho R. (2011). Intentions to Consume Omega-3 Fatty Acids: A Comparison of Protection Motivation Theory and Ordered Protection Motivation Theory. J. Diet. Suppl..

[bib0013] Ch'ng J., Glendon A. (2014). Predicting sun protection behaviors using protection motivation variables. J. Behav. Med..

[bib0014] Chamroonsawasdi K., Chottanapund S., Tunyasitthisundhorn P., Phokaewsuksa N., Ruksujarit T., Phasuksathaporn P. (2017). Development and Validation of a Questionnaire to Assess Knowledge, Threat and Coping Appraisal, and Intention to Practice Healthy Behaviors Related to Non-Communicable Diseases in the Thai Population. Behavioral Sciences.

[bib0015] Clubb A.C., Hinkle J.C. (2015). Protection motivation theory as a theoretical framework for understanding the use of protective measures [Article]. Criminal Justice Studies.

[bib0016] Cooper A.L., Brown J.A., Leslie G.D. (2021). Nurse resilience for clinical practice: An integrative review. J. Adv. Nurs..

[bib0017] Dehdari T., Hassani L., Hajizadeh E., Shojaeizadeh D., Nedjat S., Abedini M. (2014). Effects of an educational intervention based on the protection motivation theory and implementation intentions on first and second pap test practice in Iran. Asian Pacific Journal of Cancer Prevention.

[bib0018] Gaston A., Prapavessis H. (2014). Using a combined protection motivation theory and health action process approach intervention to promote exercise during pregnancy. J. Behav. Med..

[bib0019] Grace S.L., Turk-Adawi K.I., Contractor A., Atrey A., Campbell N.R., Derman W., Ghisi G.L., Sarkar B.K., Yeo T.J., Lopez-Jimenez F., Buckley J., Hu D., Sarrafzadegan N. (2016). Cardiac Rehabilitation Delivery Model for Low-Resource Settings: An International Council of Cardiovascular Prevention and Rehabilitation Consensus Statement. Progress Cardiovascular Disease.

[bib0020] Henson S., Cranfield J., Herath D. (2010). Understanding consumer receptivity towards foods and non-prescription pills containing phytosterols as a means to offset the risk of cardiovascular disease: an application of protection motivation theory [Article]. Int. J. Consum. Stud..

[bib0021] Huang C. (2012). https://search.proquest.com.

[bib0022] Joanna Briggs Institute (2020). http://joannabriggs.org/research/critical-appraisal-tools.

[bib0023] Karmakar M., Pinto S.L., Jordan T.R., Mohamed I., Holiday-Goodman M. (2017). Predicting Adherence to Aromatase Inhibitor Therapy among Breast Cancer Survivors: An Application of the Protection Motivation Theory. Breast Cancer: Basic & Clinical Research.

[bib0024] Kristoffersen A.E., Sirois F.M., Stub T., Hansen A.H. (2017). Prevalence and predictors of complementary and alternative medicine use among people with coronary heart disease or at risk for this in the sixth Tromsø study: a comparative analysis using protection motivation theory. BMC. Complement. Altern. Med..

[bib0025] Leder S., Florack A., Keller J. (2015). Self-regulation and protective health behaviour: How regulatory focus and anticipated regret are related to vaccination decisions. Psychol. Health.

[bib0026] Lynggaard V. (2019). Effects of the patient education strategy - Learning and Coping - in cardiac rehabilitation on mortality and readmissions: a randomised controlled trial (LC-REHAB). International Journal of Integrated Care (IJIC).

[bib0027] Maddux J.E., Rogers R.W. (1983). Protection Motivation and Self-Efficacy: A Revised Theory of Fear Appeals and Attitude Change. J. Exp. Soc. Psychol..

[bib0028] McGowan E., Prapavessis H. (2010). Colon cancer information as a source of exercise motivation for relatives of patients with colon cancer [Article]. Psychol. Health Med..

[bib0029] Miller S., Yardley L., Little P. (2012). Development of an intervention to reduce transmission of respiratory infections and pandemic flu: Measuring and predicting hand-washing intentions [Article]. Psychol. Health Med..

[bib0030] Mirkarimi K., Eri M., Ghanbari M.R., Kabir M.J., Raeisi M., Ozouni-Davaji R.B., Aryaie M., Charkazi A. (2017). Modifying attitude and intention toward regular physical activity using protection motivation theory: a randomized controlled trial. Eastern Mediterranean Health Journal.

[bib0031] Mirkarimi K., Mostafavi F., Eshghinia S., Vakili M.A., Ozouni-Davaji R.B., Aryaie M. (2015). Effect of Motivational Interviewing on a Weight Loss Program Based on the Protection Motivation Theory. Iran. Red. Crescent. Med. J..

[bib0032] Modeste N.N., Cort M., McLean J.E. (2018). The protection motivation theory and its impact on prostate cancer screening in Guyana. Int. Public Health J..

[bib0033] Morowatisharifabad M.A., Abdolkarimi M., Asadpour M., Fathollahi M.S., Balaee P. (2018). The Predictive Effects of Protection Motivation Theory on Intention and Behaviour of Physical Activity in Patients with Type 2 Diabetes. Open Access Macedonian Jurnal of Medical Sciences.

[bib0034] Nabizadeh S.M., Taymoori P., Hazhir M.S., Shirazi M., Roshani D., Shahmoradi B. (2018). Predicting vitamin E and C consumption intentions and behaviors among factory workers based on protection motivation theory. Environ. Health Prev. Med..

[bib0035] Nyanchoka L., Tudur-Smith C., Thu V.N., Iversen V., Tricco A.C., Porcher R. (2019). A scoping review describes methods used to identify, prioritize and display gaps in health research. J. Clin. Epidemiol..

[bib0036] Park O., Hoover L., Dodd T., Huffman L. (2011). The Use of the Modified Protection Motivation Theory to Explore Adult Consumers’ Functional Foods Consumption Behavior [In 16th Graduate Students Research Conference.

[bib0037] Park S., Kim J. (2017). Depression and problem drinking among college students in the Republic of Korea: the mediating role of protective behavioral strategies. J. Subst. Use.

[bib0038] Peixoto T.C., Begot I., Bolzan D.W., Machado L., Reis M.S., Papa V., Carvalho A.C., Arena R., Gomes W.J., Guizilini S. (2015). Early exercise-based rehabilitation improves health-related quality of life and functional capacity after acute myocardial infarction: a randomized controlled trial. Canadian Journal of Cardiology.

[bib0039] Plotnikoff R.C., Higginbotham N. (2010). Protection Motivation Theory and exercise behaviour change for the prevention of heart disease in a high-risk, Australian representative community sample of adults. Psychology. Health & Medicine.

[bib0040] Plotnikoff R.C., Lippke S., Trinh L., Courneya K.S., Birkett N., Sigal R.J. (2010). Protection motivation theory and the prediction of physical activity among adults with type 1 or type 2 diabetes in a large population sample. Br. J. Health Psychol..

[bib0041] Redd B.R. (2013). http://search.ebscohost.com/login.aspx?direct=true&db=psyh&AN=2013-99200-210&login.asp&site=ehost-live&scope=site.

[bib0042] Rogers R.W., Prentice-Dunn S. (1997). Handbook of health behavior research 1: Personal and social determinants.

[bib0043] Roth G.A., Johnson C., Abajobir A., Abd-Allah F., Abera S.F., Abyu G., Ahmed M., Aksut B., Alam T., Alam K., Alla F., Alvis-Guzman N., Amrock S., Ansari H., Arnlov J., Asayesh H., Atey T.M., Avila-Burgos L., Awasthi A., Banerjee A., Barac A., Barnighausen T., Barregard L., Bedi N., Belay Ketema E., Bennett D., Berhe G., Bhutta Z., Bitew S., Carapetis J., Carrero J.J., Malta D.C., Castaneda-Orjuela C.A, Castillo-Rivas J., Catala-Lopez F., Choi J.Y., Christensen H., Cirillo M., Cooper L., Jr, Criqui M, Cundiff D., Damasceno A., Dandona L., Dandona R., Davletov K., Dharmaratne S., Dorairaj P., Dubey M., Ehrenkranz R., El Sayed Zaki M., Faraon E.J.A., Esteghamati A., Farid T., Farvid M., Feigin V., Ding E.L., Fowkes G., Gebrehiwot T., Gillum R., Gold A., Gona P., Gupta R., Habtewold T.D., Hafezi-Nejad N., Hailu T., Hailu G.B., Hankey G., Hassen H.Y., Abate K.H., Havmoeller R., Hay S.I., Horino M., Hotez P.J., Jacobsen K., James S., Javanbakht M., Jeemon P., John D., Jonas J., Kalkonde Y., Karimkhani C., Kasaeian A., Khader Y., Khan A., Khang Y.H., Khera S., Khoja A.T., Khubchandani J., Kim D., Kolte D., Kosen S., Krohn K.J., Kumar G.A., Kwan G.F., Lal D.K., Larsson A., Linn S., Lopez A., Lotufo P.A., El Razek H.M.A., Malekzadeh R., Mazidi M., Meier T., Meles K.G., Mensah G., Meretoja A., Mezgebe H., Miller T., Mirrakhimov E., Mohammed S., Moran A.E., Musa K.I., Narula J., Neal B., Ngalesoni F., Nguyen G., Obermeyer C.M., Owolabi M., Patton G., Pedro J., Qato D., Qorbani M., Rahimi K., Rai R.K., Rawaf S., Ribeiro A., Safiri S., Salomon J.A., Santos I., Santric Milicevic M., Sartorius B., Schutte A., Sepanlou S., Shaikh M.A., Shin M.J., Shishehbor M., Shore H., Silva D.A.S., Sobngwi E., Stranges S., Swaminathan S., Tabares-Seisdedos R., Tadele Atnafu N., Tesfay F., Thakur J.S., Thrift A., Topor-Madry R., Truelsen T., Tyrovolas S., Ukwaja K.N., Uthman O., Vasankari T., Vlassov V., Vollset S.E., Wakayo T., Watkins D., Weintraub R., Werdecker A., Westerman R., Wiysonge C.S., Wolfe C., Workicho A., Xu G., Yano Y., Yip P., Yonemoto N., Younis M., Yu C., Vos T., Naghavi M., Murray C. (2017). Global, Regional, and National Burden of Cardiovascular Diseases for 10 Causes, 1990 to 2015. J. Am. Coll. Cardiol..

[bib0044] Taheri A.M., Mohebi S., Gharlipour Z. (2019). Effect of Educational Program-Based Protection Motivation Theory on Preventive Behaviors of Skin Cancer Among Farmers in Kashan. Int. J. Cancer Manage.

[bib0045] Volpe M., Battistoni A. (2018). Lifestyle and cardiovascular disease: Barefooting through the guidelines. Int. J. Cardiol..

[bib0046] Whelton P.K., Carey R.M., Aronow W.S., Casey D.E., Jr, Collins K.J., Dennison Himmelfarb C., DePalma S.M., Gidding S., Jamerson K.A., Jones D.W., MacLaughlin E.J., Muntner P., Ovbiagele B., Smith S.C., Jr, Spencer C.C., Stafford R.S., Taler S.J., Thomas R.J., Williams K.A., Sr, Williamson J.D., Wright J.T., Jr (2018). 2017 ACC/AHA/AAPA/ABC/ACPM/AGS/APhA/ASH/ASPC/NMA/PCNA Guideline for the Prevention, Detection, Evaluation, and Management of High Blood Pressure in Adults: A Report of the American College of Cardiology/American Heart Association Task Force on Clinical Practice Guidelines. J. Am. Coll. Cardiol..

[bib0047] Whittemore R., Knafl K., 52 (2005). The integrative review: updated methodology. J. Adv. Nurs..

[bib0048] Wong T.S., Gaston A., DeJesus S., Prapavessis H. (2016). The utility of a protection motivation theory framework for understanding sedentary behavior. Health Psychol. Behav. Med..

[bib0049] Zhang L., Li X., Zhou Y., Lin D., Su S., Zhang C., Stanton B. (2015). Predictors of Consistent Condom Use Among Chinese Female Sex Workers: An Application of the Protection Motivation Theory. Health Care Women. Int..

[bib0050] Zhang Y., Cooke R. (2012). Using a combined motivational and volitional intervention to promote exercise and healthy dietary behaviour among undergraduates. Diabetes Res. Clin. Pract..

